# An analysis of electroacupuncture as adjunctive treatment for insomnia: a new perspective targeting GABA-mediated microbiome-gut-brain axis

**DOI:** 10.3389/fneur.2025.1504316

**Published:** 2025-04-30

**Authors:** Lijuan Yan, Xiao Wang, Xiang Liu, Jiaying Cai, Zuobing Zhang, Bin Yang

**Affiliations:** ^1^Department of Anesthesiology, The First Affiliated Hospital of Xiamen University, School of Medicine, Xiamen University, Xiamen, China; ^2^Department of Ultrasound, The First Affiliated Hospital of Xiamen University, School of Medicine, Xiamen University, Xiamen, China; ^3^Department of Physiology, Michigan State University, East Lansing, MI, United States; ^4^Department of Scientific Research, The First Affiliated Hospital of Xiamen University, Xiamen, China

**Keywords:** insomnia, electroacupuncture, γ-aminobutyric acid, gut microbiota, microbiome-gut-brain axis

## Abstract

**Introduction:**

Insomnia is a prevalent psychiatric disorder that significantly impacts mental and physical health. Emerging evidence suggests that gut microbiota, particularly γ-aminobutyric acid (GABA) producing bacteria, plays a critical role in the microbiome-gut-brain axis (MGBA), influencing sleep and mental states. Electroacupuncture (EA) has been shown to have favorable anti-insomnia effects. This research protocol outlines a randomized controlled trial (RCT) designed to investigate the efficacy of EA in modulating GABA levels via the MGBA to alleviate insomnia.

**Methods and analysis:**

The study will use glutamate (Glu) concentrations of p-chlorophenylalanine (PCPA) -induced insomnia rat model to examine whether EA at specific acupoints can increase GABA levels in the brain and plasma by enhancing the abundance of GABA-producing gut bacteria. A second experiment will transplant stool from insomnia rats into germ-free mice to evaluate the causal role of microbiota in insomnia pathology. Primary outcome measures include changes in GABA and Glu levels, data about the open field test, and gut microbiota composition.

**Discussion:**

This study will provide insights into the therapeutic mechanisms of EA targeting the MGBA in the treatment of insomnia and contribute to the development of novel therapeutic strategies.

**Ethics and dissemination:**

This study has been approved by the Laboratory Animal Center of Xiamen University (No. XMULAC20220100). The study findings will be submitted for publication in peer-reviewed academic journals.

## Graphical abstract

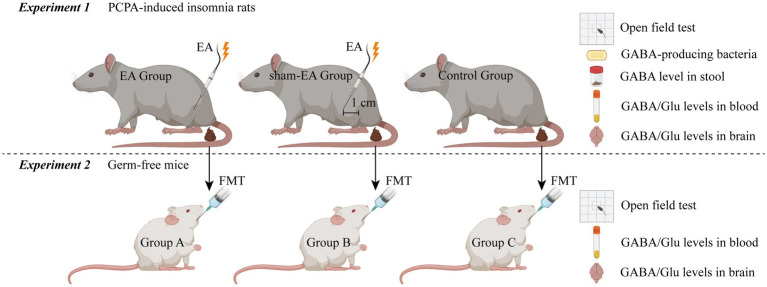

PCPA, p-chlorophenylalanine; EA, Electroacupuncture; FMT, Fecal Microbiota Transplantation; GABA, γ-aminobutyric acid; Glu, glutamate. Created in BioRender. Liu, X. (2025) https://BioRender.com/pejhdmp.

## 1 Introduction

Insomnia is a prevalent psychiatric disorder that can have negative effects on both physical and psychological health, ultimately reducing the quality of life (1–3). Epidemiological studies suggest that insomnia affects approximately 6–15% of individuals (4, 5). In recent years, the impact of the coronavirus disease (COVID-19) on mental state, health anxieties, and social interaction has been an undeniable focus point (6, 7). Persistent perceived stress, hyperarousal states, and Post-Traumatic Stress Disorder (PTSD) symptoms during the pandemic have physiologically interfered with sleep architecture (8). Moreover, emerging research has indicated that substantial alterations in brain structure are present in these patients, affecting different lobes and associated with the quality of sleep (9). Standard long-term pharmacological treatments, though effective initially, often result in tolerance, dependence, withdrawal symptoms, and rebound insomnia, along with adverse effects such as neurotoxicity and disturbances in sleep architecture (10–12). Clinical guidelines recommend cognitive behavior therapy for insomnia (CBT-I) as a first-line treatment (11). However, its widespread implementation in clinical practice is impeded by high costs and a shortage of qualified psychotherapists (13). These challenges highlight the urgent need for alternative, accessible, and effective therapeutic approaches.

Electroacupuncture (EA), a technique that combines traditional acupuncture with electrical stimulation, has emerged as a promising non-pharmacological treatment for insomnia through multi-targeted and multi-level mechanisms (such as influencing neurotransmitter levels, hormone regulation, and gut microbiota composition). This assertion is supported by evidence of significant improvements in objective sleep maintenance and architecture (3, 14–19). Specifically, EA has been shown to modulate key neurotransmitters such as γ-aminobutyric acid (GABA) and glutamate (Glu), both of which play essential roles in regulating the sleep–wake cycle. Despite these positive results, the underlying precise molecular mechanisms by which EA exerts its anti-insomnia effects have not been fully elucidated (1, 20, 21).

### 1.1 The impact of gut microbiota on sleep and mental state

Recent research has emphasized the role of gut microbiota in regulating sleep and mental health (22). A balanced gut microbiota composition is crucial for normal brain function and mental well-being, and disturbances in the gut microbiota (dysbiosis) have been implicated in both insomnia and various mental disorders, such as depression (23–25). The gut microbiota influences brain function via multiple pathways, including the immunoregulatory, neuroendocrine, and vagus nerve pathways (24). Gut bacteria such as *Bacteroides*, *Parabacteroides*, and *Escherichia* have been shown to produce GABA, a key neurotransmitter involved in sleep regulation (26). Alterations in GABAergic signaling have been linked to both sleep disturbances and mood disorders, including depression (24, 27). Furthermore, studies have suggested that supplementing GABA-producing bacteria can alter GABA receptor expression in the brain and reduce depression-related behaviors in animal models (28). Additionally, similar studies have found that GABA supplementation via fermented foods improves sleep quality and reduces anxiety in both mice and humans (25, 29, 30).

### 1.2 Regulation mechanism of EA for anti-insomnia

Acupuncture has been used in China for centuries to treat primary insomnia. Emerging studies indicated that the microbiome-gut-brain axis (MGBA) has emerged as a potential therapeutic target for insomnia (31, 32). The mechanisms of the MGBA, including the vagus nerve, the prevertebral sympathetic ganglia, and the hypothalamic–pituitary–adrenal (HPA) axis, may be significant pathways influenced by GABA (33). The gut microbiome plays a critical role in regulating the HPA axis through the production of neuroactive metabolites and the modulation of neurotransmitter and cytokine synthesis. This includes the synthesis of GABA, norepinephrine, serotonin, acetylcholine, and dopamine, which occur at local, systemic, and central levels (34, 35).

Evidence suggests that EA can reshape intestinal microflora structure and enhance diversity within the intestinal flora, resulting in inhibiting the peripheral and central nerve system inflammatory response (23, 36–38). The mediated role of the HPA axis may be implicated in this phenomenon. Sleep deprivation is frequently regarded as a physiological stressor. It can activate the HPA axis and induce increased permeability of the gut, thereby facilitating the passage of bacteria and bacterial antigens across the epithelial barrier (39, 40). EA can act on the center of the sympathetic nervous control center, thereby reducing oxidative stress, regulating sympathetic excitability, and normalizing cortisol levels (14, 41). Consequently, EA may create a favorable environment for microbial diversity by reducing stress-induced dysbiosis.

Furthermore, EA also exerts its anti-insomnia effects by influencing various neurotransmitters, such as endogenous melatonin, inflammatory mediators, short-chain fatty acids (SCFAs), lipopolysaccharides (LPS), dopamine, brain-derived neurotrophic factor (BDNF), as well as GABA and glutamate in the central nervous system (3, 23, 42–44).

GABA has been demonstrated to play a pivotal role in regulating the sleep–wake cycle. Both GABA-producing bacteria and direct GABA supplementation have shown anti-insomnia effects. Evidence from experimental models of mice has shown that EA can increase GABA levels in both the hypothalamus and the peripheral blood, and this increase may be related to the vagus nerve stimulated by EA (45). EA has been demonstrated to promote the expression of glutamic acid decarboxylase 67 (GAD67), which in turn up-regulates GABA expression, thereby inhibiting excitatory postsynaptic potentials. Additionally, EA has been shown to promote GABA and its receptors (GABA-AR, GABA-BR), while concurrently inhibiting the release of excitatory neurotransmitters, such as glutamate (46). However, it is important to note that these mechanisms were validated in the pain model, not the insomnia model. Moreover, A reduction in the levels of GABA is a distinctive marker of objective sleep disorders, which is linked to alterations in the composition of intestinal microorganisms and short-chain fatty acids. Lactic acid bacteria and bifidobacteria are the primary intestinal bacteria that produce GABA (47). However, it remains unclear whether the mechanism of EA for anti-insomnia is mediated through changes in the abundance of GABA-producing bacteria in the gut. Consequently, further exploration is necessary to elucidate the fundamental underlying mechanism, a necessity which will be addressed in subsequent studies.

### 1.3 Hypothesis: EA modulates GABA-producing gut microbiota to alleviate insomnia via MGBA

Current research indicates a correlation between EA, insomnia, depression, and dysbiosis of the gut microbiota (33). Our perspective proposes that EA may target GABA-producing microbiota to increase plasma and brain GABA levels, thereby alleviating insomnia through MGBA, building on three key lines of evidence: (i) EA’s documented effects on gut microbiota: Prior studies demonstrate that EA can modulate gut microbial diversity and composition in conditions such as Parkinson’s disease and postoperative cognitive dysfunction, though not yet specifically in insomnia models (36, 48). (ii) GABA-producing bacteria and sleep: Gut bacteria (e.g., Lactobacillus, Bifidobacterium) are known to synthesize GABA, and their supplementation improves sleep quality in preclinical models (25, 26, 49, 50). (iii) EA’s regulation of GABAergic signaling: EA increases GABA levels in the hypothalamus and plasma in insomnia models, likely via vagal stimulation and upregulation of glutamic acid decarboxylase (GAD67) (45).

Of course, it is critical to acknowledge the current lack of direct evidence linking EA to specific GABA-producing bacterial taxa in insomnia models. Therefore, the article further introduces a novel conceptual roadmap for future experimental studies. By integrating microbial sequencing, neurotransmitter profiling, and Fecal Microbiota Transplantation (FMT)-based causality testing, we aim to either validate this hypothesis or identify alternative mechanisms underlying EA’s efficacy.

## 2 Methods and analysis

### 2.1 Study design

**Figure 1:**
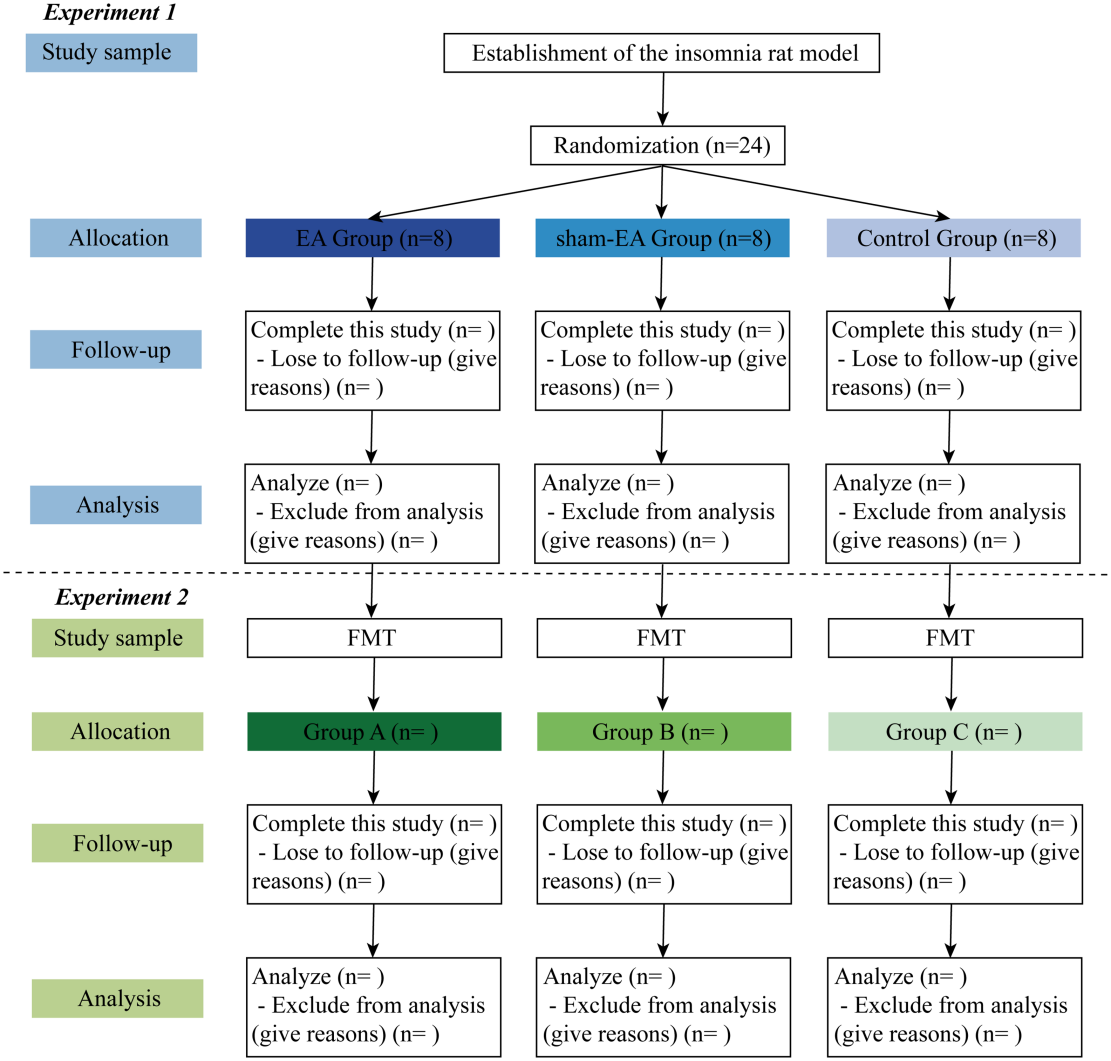
The study flow chart. EA, Electroacupuncture; FMT, Fecal Microbiota Transplantation.

**Figure 2:**
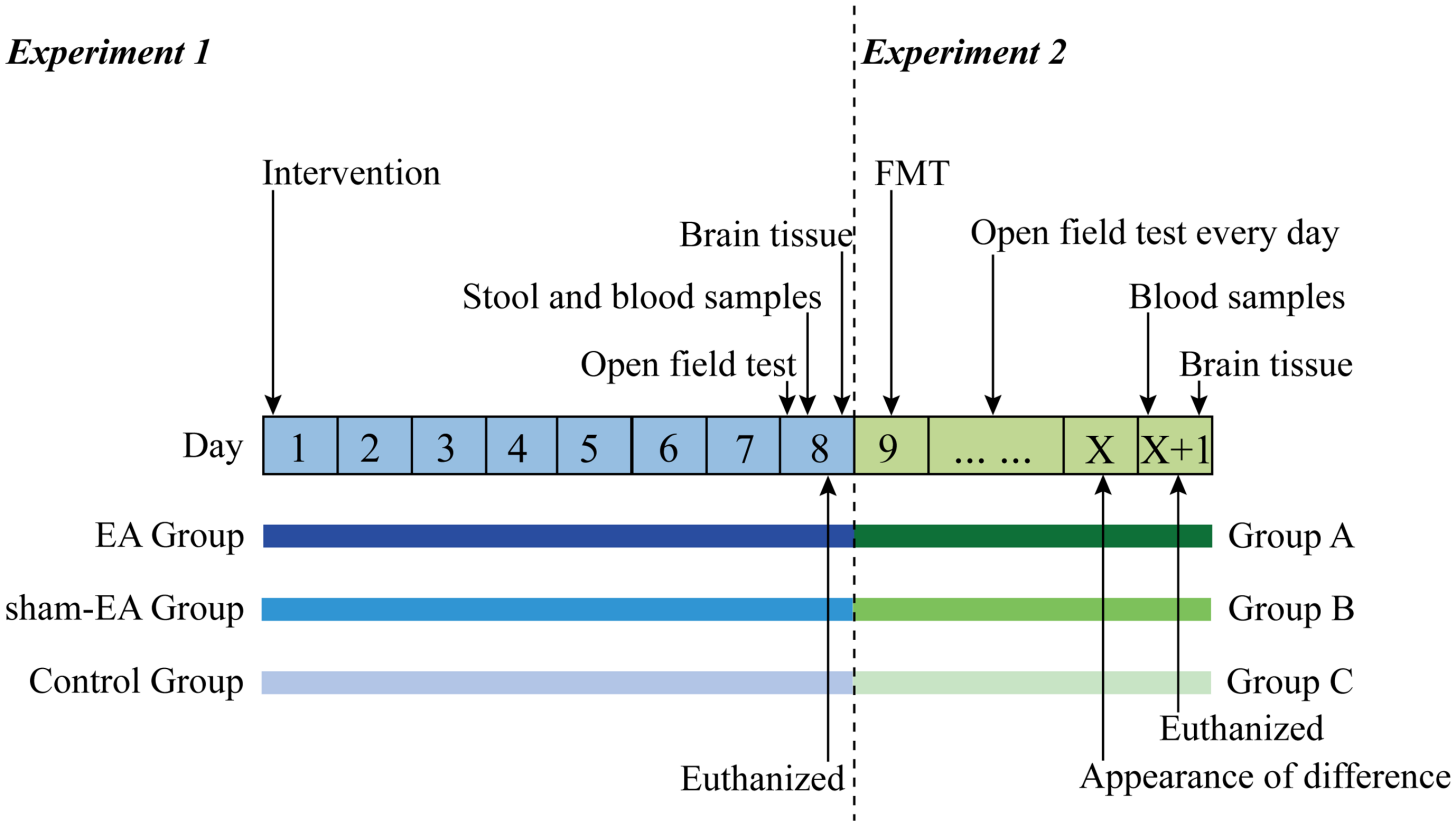
The detailed timeline diagram of model establishment, group allocation, interventions, sample collection, and sample measurement. EA, Electroacupuncture; FMT, Fecal Microbiota Transplantation.

This is a double-blinded RCT scheduled to be conducted in the laboratory of the First Affiliated Hospital of Xiamen University. The study will follow all related animal experience rules and the study flow chart (Figure 1). The study will be divided into two sections, as illustrated in the graphical abstract (Graphical abstract). Once the insomnia rat model has been established, this study will follow the detailed timeline diagram of model establishment, group allocation, interventions, sample collection, and sample measurement shown in Figure 2.

#### 2.1.1 Experiment 1

Twenty-four rats with PCPA-induced insomnia will be randomly assigned to one of three groups in a 1:1:1 ratio: the EA group (*n* = 8), the sham-EA group (*n* = 8), and the control group (*n* = 8). The EA group will receive EA intervention at designated acupoints, while the sham-EA group will receive EA at sham acupoints located 1 cm away from the corresponding acupoints. The control group will not receive EA. All rats will undergo the open field test and will eventually be euthanized. Stool, blood, and brain samples will be collected following standard operation specifications.

#### 2.1.2 Experiment 2

Germ-free mice will be administered with insomnia rats’ stool via intragastric administration. Subsequently, all mice will undergo the open field test and will eventually be euthanized. Blood and brain samples will be collected following standard operation specifications.

### 2.2 Study sample

#### 2.2.1 Establishment of the insomnia rat model

Adult male Sprague–Dawley rats (weighing 320 ± 20 g; Harlan, Indianapolis, IN) will be intraperitoneally injected with PCPA at a dosage of 300 mg·kg^−1^·d^−1^ for 2 consecutive days. After 28–30 h, observable physical symptoms such as disheveled hair, significant weight loss, and irritability will appear, indicating successful modeling (51, 52).

#### 2.2.2 Sample size calculation

Under the premise of adhering to the 3R principle of experimental animals and minimizing the experimental animal loss, twenty-four rats with PCPA-induced insomnia will be used in this study. Each group will consist of eight rats.

#### 2.2.3 Randomization

Rats with PCPA-induced insomnia will be randomly assigned to one of three groups using a computer-generated randomized sequence. The randomization results will be concealed in opaque envelopes.

#### 2.2.4 Blind

All rats and mice will be distinguished by ear tag before the intervention process. An independent research assistant will carry out all aspects of the intervention process, including EA intervention and stool transplantation. Another independent research assistant, who is blinded to the group allocation, will collect data from the open field test, collect samples, and test samples. The biostatistician will receive anonymized datasets labeled only by group codes to maintain blinding during analysis.

### 2.3 Intervention

#### 2.3.1 Experiment 1: EA intervention for rats with PCPA-induced insomnia

Rats with PCPA-induced insomnia will be housed under standard laboratory conditions at a temperature of 25 ± 1°C and a humidity of 50 ± 5%. They will be provided with a standard diet and water (51) and exposed to a 12-h alternate light–dark cycle throughout the experimental period. The EA group will receive EA stimulation at Baihui, Sanyinjiao, and Shenmen acupoints once daily for seven consecutive days. The electrical stimulation will last for 15 min at an alternating frequency of 2/15 Hz and an intensity of 1 mA each time (53, 54). For the sham-EA group, rats will receive EA at sham acupoints located 1 cm away from the corresponding acupoints. The control group will not receive EA.

#### 2.3.2 Experiment 2: transplant insomnia rats’ stool into germ-free mice

Insomnia rats’ stools will be transplanted into 24 male C57BL/6J mice of SPF grade (weight 20 ± 2 g) separately. All mice will be housed in sterilized isolators at a temperature of 25 ± 1°C and a humidity of 50 ± 5%. The mice will be fed a standard diet and provided with water. The experimental period will involve exposing the mice to a 12-h alternate light–dark cycle.

### 2.4 Sample collection and measurement

#### 2.4.1 Experiment 1

After a 7-day intervention based on group allocation, all rats will undergo an open field test. The test will be conducted one by one following a 10-min acclimation period to the environment. Exercise distance, exercise time, and number of arm liftings will be calculated by the video tracking analysis system within 5 min (21, 51).

On the day of the open field test, stool will be collected and immediately placed into pre-coded sterile containers. The stool will then be stored at −80°C and subjected to DNA isolation within 30 min after collection. The 16S rDNA sequencing will be performed to analyze within 2 weeks after collection (55). Simultaneously, the same samples will be dissolved and centrifuged for 20 min at 4°C and 3,000 r/min. The supernatant will then be collected. The levels of GABA in the stool samples will be measured strictly following the instructions provided by the ELISA kit.

On the day of the open field test, blood samples will be collected from the femoral vein under ultrasound guidance and left for 1 h. Serum samples will be separated by 10 min of centrifugation at 4°C and 3,000 r/min (51). The levels of GABA and Glu in the blood samples will be measured strictly following the instructions provided by the ELISA kit.

Following the collection of stool and blood samples, the rats will be euthanized through cervical dislocation. The skull will then be removed to expose the brain tissue. The hypothalamus and brainstem tissues will be rapidly dissected, weighed, wrapped in tin foil, placed in an ice bath, and then quickly transferred to liquid nitrogen for storage at −80°C (51). The levels of GABA and Glu in the brain samples will be measured strictly following the instructions provided by the ELISA kit.

#### 2.4.2 Experiment 2

Following fecal transplantation, the mice will undergo daily open field tests until differences among groups become apparent. Subsequently, blood and brain samples will be collected and stored as described in Experiment 1. Additionally, the levels of GABA and Glu in the plasma and brain will be measured meticulously.

## 3 Outcome measurements

### 3.1 Experiment 1

- Data about the open field test: exercise distance (measured in centimeters), exercise time (measured in seconds), and number of arm liftings.
- Data on gut microbial profiles of stool samples.
- The levels of GABA in stool.
- The levels of GABA and Glu in plasma and brain samples, as well as the ratio of GABA to Glu.

### 3.2 Experiment 2

- Data about the open field: exercise distance, exercise time, and number of arm liftings.
- The levels of GABA and Glu in plasma and brain samples, as well as the ratio of GABA to Glu.

### 3.3 Statistical analysis

All statistical analyses will be conducted utilizing the Statistical Product and Service Solutions statistical software (Version 20.0) and R software (Version 4.3.2) programs. Continuous variables will first undergo a series of normality tests, including the Shapiro–Wilk test for assessing data distribution and the Levene’s test for evaluating the variability of the variances.

#### 3.3.1 Experiment 1

Behavioral outcomes (open field test): One-way ANOVA (for normally distributed data) or Kruskal-Wallis test (non-parametric) will be employed to compare exercise distance, time, and arm liftings across the EA, sham-EA, and control groups. Post-hoc comparisons (Tukey’s HSD test) will adjust for multiple comparisons.

GABA/Glu levels: Linear mixed-effects models will account for repeated measures (e.g., plasma vs. brain GABA) with group, time, and tissue type as fixed effects.

Gut microbiota: Alpha diversity (Shannon index) and beta diversity (Bray–Curtis dissimilarity) will be calculated using Quantitative Insights Into Microbial Ecology 2 (QIIME2). Permutational Multivariate Analysis of Variance (PERMANOVA) (999 permutations) will be utilized to test for group differences in microbial composition. The differential abundance of GABA-producing taxa will be identified using DESeq2.

Finally, the study will also assess the correlations between the various parameters. Spearman’s rank correlation will assess relationships between microbial abundance, GABA/Glu ratios, and behavioral outcomes.

#### 3.3.2 Experiment 2

FMT effects: A repeated-measures ANOVA (with Greenhouse–Geisser correction if sphericity is violated) will analyze open field outcomes over time. A two-way ANOVA will be employed to compare post-FMT GABA/Glu levels between recipient mice that have been transplanted with microbiota that has been treated with EA as opposed to untreated microbiota.

Causal inference: Mediation analysis (PROCESS macro) will evaluate whether microbiota shifts mediate EA’s effects on GABAergic signaling.

The effect sizes and 95% confidence intervals will be reported. A significance threshold of *p* < 0.05 (two-tailed) will be applied, with False Discovery Rate (FDR)-adjusted q-values for microbiota analyses.

## 4 Discussion

Insomnia can have negative effects on both physical and mental well-being. Therefore, it is important to focus on anti-insomnia strategies. This article initially outlines the persuasive evidence regarding the effects of gut microbiota on sleep and mental state and discusses the possible regulation mechanism of EA for anti-insomnia. This article presents a new perspective: GABA may be a direct target of EA for treating insomnia, functioning as a key mediator regulating MGBA. By combining FMT with germ-free models and correlating GABA levels with microbial shifts, we aim to establish causality between EA-induced microbiota changes and insomnia relief. If confirmed, this mechanism could advance EA as a microbiota-targeted therapy for sleep disorders. If not, our findings may instead highlight alternative pathways (e.g., HPA axis modulation, neurotransmitter regulation) as primary mechanisms.

Given that gut microbiota is influenced by a multitude of factors, including diet, genetics, environment, and medication use, the rigorous experimental design employed in this study allows for the isolation of specific effects of EA on gut microbiota modulation through the following four aspects. (i) Strengthened control groups. To explicitly differentiate EA-specific effects from non-specific interventions or environmental influences, we have incorporated three distinct control groups in Experiment 1. By comparing outcomes across these groups, we can isolate effects attributable to acupoint specificity (EA vs. sham-EA) and intervention presence (sham-EA vs. untreated control). (ii) Standardization of environmental and procedural variables. To minimize confounding variables, the housing conditions, handling procedures, and timing of interventions of the three groups will be handled identically. The implementation of blinding is also a measure that can be adopted to mitigate the potential for observer bias. (iii) Donor specificity and recipient controls. Experiment 2 has the potential to establish causality between EA-induced microbiota changes and insomnia relief. (iv) Statistical and analytical rigor. Confounder adjustment and causal mediation analysis are included in this study design.

EA at specific acupoints may be more effective for treating insomnia than the current commonly prescribed method of using hypnotics and other interventions. The present study has demonstrated that EA exerts a favorable therapeutic effect on insomnia, without serious adverse reactions. This is in contrast to the potential consequences of long-term anti-insomnia medication, which can result in drug dependence and resistance. Simultaneously, EA has been shown to effectively alleviate the effect of zolpidem on sleep quality in patients with insomnia following drug discontinuation (15, 56, 57). Consequently, EA is deserving of clinical utilization and advocacy. Furthermore, although there is an increasing body of evidence supporting the use of EA for sleep and emotional disorders, such as insomnia, stress, anxiety, and depression, through MGBA, further controlled, reproducible studies are necessary to reach definitive conclusions regarding the biological significance of EA on the mechanism of anti-insomnia and to determine the comparative efficacy of EA to other therapeutic interventions.

It is imperative to investigate the mechanisms by which EA communicates with MGBA based on GABA-producing microbiota for the advancement of insomnia therapeutic strategies. The study aims to investigate whether EA relies on higher levels of GABA-producing bacteria to increase plasma and brain GABA levels, thus effectively treating insomnia. Compelling preclinical evidence further demonstrates that manipulating the stool microbiome can alter plasma GABA levels, which can help regulate the host’s sleep and mental state (1, 24, 27). EA has the potential to increase the abundance of GABA-producing microbiota, thus resulting in inflammation reduction and fatigue mitigation. It can also regulate the balance of GABA and Glu in the brain. It is important to note that GABA has indirect effects on the nervous system, which are mediated through the gut due to its inability to pass through the blood–brain barrier (BBB) (25). The impact of EA on GABA levels can be either transient or enduring. The transient effect of EA may be attributable to its rapid regulation of GABA release, rendering it a suitable intervention for the alleviation of acute symptoms, such as acute pain or stress (58). When EA maintains the inhibitory tension of GABA energy by enhancing receptor sensitivity or improving salient plasticity, its cumulative effect is more significant, which is suitable for the management of chronic diseases (e.g., insomnia and depression) (53). Subsequent studies will provide further evidence regarding the timeliness of the effect of EA.

### 4.1 Limitations and future directions

The present study focuses on the interactions between GABAergic signaling and the MGBA. However, emerging evidence suggests that microglial activation may be a critical mediator of sleep-microbiota crosstalk (59, 60). In neurodegenerative disorders, gut dysbiosis has been shown to induce microglial senescence and neuroinflammation, characterized by dystrophic morphology, which may similarly disrupt sleep architecture in insomnia (59–62). Notably, microbiome transplantation in depression models (63) and probiotic interventions in Parkinson’s disease (64) have been shown to reduce pro-inflammatory cytokines (e.g., IL-1β, TNF-α) and normalize microglial activity. These findings raise the possibility that EA or GABA-producing microbiota transplantation could attenuate insomnia-related neuroinflammation by shifting microglial states (e.g., from amoeboid to ramified morphology). Further research integrating microglial profiling (e.g., Iba1 immunohistochemistry, senescence markers) with microbiota sequencing is required to test this hypothesis and unify the MGBA signaling with neuroimmune pathways in sleep regulation.

Previous studies have shown that several acupoints, such as Baihui, Shenting, Neiguan, Shenmen, and Sanyinjiao, are commonly used to treat insomnia (53, 54, 65, 66). However, based on our previous study experience, we have selected the Baihui, Sanyinjiao, and Shenmen acupoints for this study protocol. It is important to note that the efficacy of anti-insomnia may differ on other acupoints. In further research, it is necessary to determine which acupoints can effectively regulate the abundance of GABA-producing microbiota in the gut. To obtain more evidence on the ideal duration, cycle, and intensity of EA in rats, larger and more rigorously designed trials should be encouraged. Additionally, long-term studies will also clarify whether EA-induced microbiota changes are sustained and predictive of lasting sleep improvement.

Lastly, although our preclinical study combines controlled experiments, microbiome sequencing, and FMT to rigorously test causal links between EA, gut microbiota, and insomnia relief, translational human trials are needed to confirm clinical relevance. Future work will integrate multi-omics (metagenomics/metabolomics) and standardized EA protocols to identify conserved microbial and neurochemical signatures across species.

## 5 Conclusion

Elucidating the underlying molecular mechanisms of EA as an anti-insomnia therapy constitutes a paradigm shift in sleep research. It not only enhances our comprehension of insomnia’s pathogenesis but also provides the potential to uncover novel targets for sleep intervention, ultimately facilitating the optimization of insomnia treatment.

## Author’s note

For technical assistance, contact Lijuan Yan.
